# Incidence and predictors of failed second-generation endometrial ablation

**DOI:** 10.1186/s10397-017-1030-4

**Published:** 2017-12-15

**Authors:** Jordan Klebanoff, Gretchen E. Makai, Nima R. Patel, Matthew K. Hoffman

**Affiliations:** 1 Department of Obstetrics & Gynecology, Christiana Care Health System, 4755 Ogletown-Stanton Road, Suite 1905, Newark, DE 19718 USA; 2 Division of Minimally Invasive Gynecology, Christiana Care Health System, Newark, DE USA

**Keywords:** Endometrial ablation, Treatment failure, Hysterectomy, Risk factors

## Abstract

**Background:**

The need for any treatment following an endometrial ablation is frequently cited as “failed therapy,” with the two most common secondary interventions being repeat ablation and hysterectomy. Since second-generation devices have become standard of care, no large cohort study has assessed treatment outcomes with regard to only these newer devices. We sought to determine the incidence and predictors of failed second-generation endometrial ablation, defined as the need for surgical re-intervention.

We performed a retrospective cohort study at a single academic-affiliated community hospital. Subjects included women undergoing second-generation endometrial ablation for benign indications between October 2003 and March 2016. Second-generation devices utilized during the study period included the radiofrequency ablation device (RFA), hydrothermal ablation device (HTA), and the uterine balloon ablation system (UBA).

**Results:**

Five thousand nine hundred thirty-six women underwent endometrial ablation at a single institution (3757 RFA (63.3%), 1848 HTA (31.1%), and 331 UBA (5.6%)). The primary outcome assessed was surgical re-intervention, defined as hysterectomy or repeat endometrial ablation. Of the total 927 (15.6%) women who required re-intervention, 822 (13.9%) underwent hysterectomy and 105 (1.8%) underwent repeat endometrial ablation. Women who underwent re-intervention were younger (41.6 versus 42.9 years, *p* < .001), were more often African-American (21.8% versus 16.2%, *p* < .001), and were more likely to have had a primary radiofrequency ablation procedure (hazard ratio 1.37; 95%CI 1.01 to 1.86). Older age was associated with decreased risk for treatment failure with women older than 45 years of age having the lowest risk for failure (*p* < .001). Age between 35 and 40 years conferred the highest risk of treatment failure (HR 1.59, 95% CI 1.32–1.92). Indications for re-intervention following ablation included menorrhagia (81.8%), abnormal uterine bleeding (27.8%), polyps/fibroids (18.7%), and pain (9.5%).

**Conclusion:**

Surgical re-intervention was required in 15.6% of women who underwent second-generation endometrial ablation. Age, ethnicity, and radiofrequency ablation were significant risk factors for failed endometrial ablation, and menorrhagia was the leading indication for re-intervention.

## Background

Endometrial ablation, a surgical procedure to decrease or control heavy menstrual bleeding, is generally intended for premenopausal women who have failed, or are not candidates for, medical therapy. Ablation is contraindicated in women with undiagnosed abnormal bleeding and those who desire future fertility [1]. While hysteroscopic resection and ablation became the gold standard for endometrial ablation in the 1980s and 1990s, “second-generation” endometrial ablation devices were developed in the late 1990s and early 2000s in order to improve ease, safety, and uniformity of ablation procedures [2]. The uterine balloon ThermaChoice® (UBA) (Gynecare, Somerville, New Jersey) received its first FDA approval in 1997, while the HydroThermAblator® hydrothermal ablation (HTA) (Boston Scientific, Marlborough, Massachusetts) and NovaSure® radiofrequency ablation (RFA) (Hologic Inc., Marlborough, Massachusetts) devices gained their approvals in 2001 [3].

The need for any treatment following an endometrial ablation is frequently cited as “failed therapy,” with the two most common secondary interventions being repeat ablation and hysterectomy [4–10]. In a longitudinal 5-year follow-up study of 139 women randomized to either RFA or HTA, rates of subsequent surgery were high (24% of the entire group) while relative risk was 0.43 for women in the RFA group compared to HTA [11]. The majority of the women who underwent subsequent surgery had a hysterectomy, as opposed to repeat ablation.

Surgical re-intervention rates above 20% suggest there is opportunity to improve outcomes of the primary intervention; one approach would be to improve patient selection for ablation. Risk factors previously identified for failed endometrial ablation include younger age at initial procedure, history of cesarean delivery, tubal ligation, and abnormal uterine findings on radiologic assessment including leiomyoma, thickened endometrial stripe, and polyps [5, 6, 8]. Since second-generation devices have become standard of care, no large cohort study has assessed treatment outcomes with regard to only these newer devices. The purpose of this study is to establish the rate of failed second-generation endometrial ablation, defined as subsequent hysterectomy or repeat ablation, in a large US-based cohort.

## Methods

After obtaining approval by the Institutional Review Board, we performed a retrospective cohort study of women who had undergone an endometrial ablation from October 2003 through March 2016. Patients were identified using a contemporaneous electronic database. Data was extracted by using relevant International Classification of Diseases—Ninth revision (ICD-9) codes as well as Current Procedural Terminology (CPT) codes. Women were included if they had undergone RFA, HTA, or UBA for benign indications at a single academic-affiliated community hospital (choice of device utilized was based on physician preference).

Women were excluded if they had a diagnosis related to any gynecologic malignancy; if the ablation was performed by any modality other than RFA, HTA, or UBA; or if the indication for the ablation was post-menopausal bleeding.

The initial cohort of women identified was then re-analyzed, using relevant ICD-9 and CPT codes, to isolate any patient who underwent either a hysterectomy or repeat endometrial ablation at a date after their initial endometrial ablation. Subjects’ electronic health records were further analyzed by reviewing operative reports to verify successful performance of the initial endometrial ablation procedure as well as any re-intervention. Women whose index ablation or subsequent re-intervention could not be confirmed in detailed operative reports were excluded.

The primary outcome evaluated was the incidence of surgical re-intervention, defined as either hysterectomy or repeat ablation. Exposures examined include influence of age, body mass index, race/ethnicity, ablation device type, concomitant or history of tubal ligation, indication for endometrial ablation, and concomitant uterine or adnexal surgery. All pertinent patient data were either extracted from the electronic health record or identified by review of operative reports. The data was initially analyzed using univariate modeling, using Stata 14.0 (College Station, TX). Recognizing that different ablation technologies were popularized at different time periods, Cox proportional hazard testing was used to adjust for the time to failure.

## Results

**Fig. 1:**
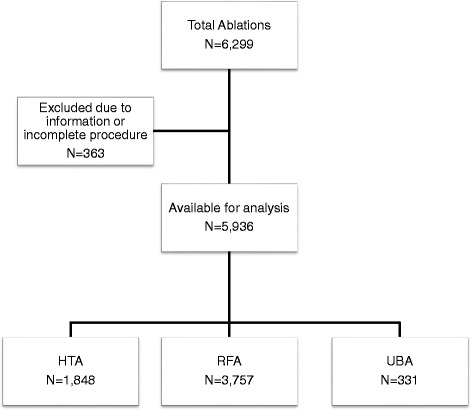
Consort diagram of patient flow

Between October 2003 and March 2016, we identified 6299 women who underwent an endometrial ablation at a single academic-affiliated community hospital. After excluding 363 women due to missing information from the electronic health record or failure to undergo their scheduled ablation, 5936 women were eligible for analysis. Procedure distribution was as follows: 3757 RFA (63.3%), 1848 HTA (31.1%), and 331 UBA (5.6%) (Fig. 1). The mean age (± standard deviation) was 42.7 ± 5.7 years. The mean BMI was 29.9 ± 7.8 kg/m^2^. The majority of women included were Caucasian (79.3%), with the remainder predominantly African-American (17%) (Table 1).

**Table 1:** Univariable analysis of risk factors for re-intervention

Variable	Re-intervention (%)	No Re-intervention (%)	*p* value
Total patients	927 (15.6)	5009 (84.4)	
Age (years)			
Mean	41.59	42.94	< .001
Age band < 35	90 (9.7)	388 (7.8)	
35–40	223 (24.1)	908 (18.1)	
40–45	324 (35.0)	1647 (32.9)	
45–50	233 (25.1)	1516 (30.3)	
> 50	57 (6.2)	550 (11.0)	
Race			
Asian	3 (0.3)	35 (0.7)	< .001
Black	202 (21.8)	809 (16.2)	
White	685 (73.9)	4020 (80.3)	
Other	37 (4.0)	145 (2.9)	
Weight (kg)	80.84	80.71	.60
Height (cm)	163.70	164.33	.07
BMI (kg/m^2^)			
Mean	30.17	29.89	.75
< 18	1 (9.1)	10 (90.9)	
18–25	95 (10.3)	830 (89.7)	
25–30	113 (11.8)	845 (88.2)	
30–35	90 (13.9)	556 (86.1)	
35–40	46 (10.9)	375 (89.1)	
> 40	35 (11.2)	279 (88.9)	
Ablation technique			
Uterine balloon	47 (14.2)	284 (85.8)	
Hydrothermablation	349 (18.9)	1499 (81.1)	
Radiofrequency	531 (14.1)	3226 (85.9)	< .001

**Fig. 2:**
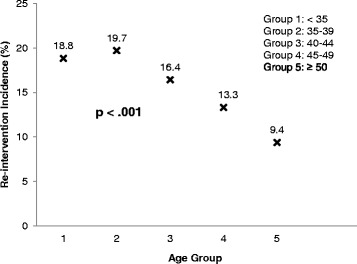
Incidence of re-intervention by age grouping

Surgical re-intervention was required in 927 women in the cohort (15.6%). Hysterectomy was performed in 822 women (13.8%) and endometrial ablation in 105 women (1.8%). Women who were younger and women who were African-American were more likely to require re-intervention (Table 1). Subjects were further stratified into five groups based on age at initial ablation: group 1, age < 35 years; group 2, age 35–39 years; group 3, age 40–44 years; group 4, age 45–49 years; and group 5, age ≥ 50 years (Fig. 2). Women in groups 1, 2, and 3 were more likely to require re-intervention compared to women in groups 4 and 5 (*p* < .001). The incidence of re-intervention for women in groups 1, 2, and 3 were 18.8, 19.7, and 16.4% respectively. Women in group 2 (age 35–39) had the highest likelihood of re-intervention (HR 1.59, 95% CI 1.32–1.92). Neither BMI, nor BMI category, affected the rates of re-intervention. Main indications for surgical re-intervention following ablation were menorrhagia (81.8%), abnormal uterine bleeding (27.8%), polyps or fibroids (18.7%), and pain (9.5%) (note: patients could have more than one diagnosis listed).

**Fig. 3:**
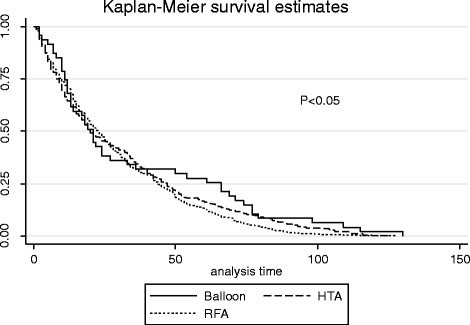
Kaplan-Meier survival curve estimates

**Fig. 4:**
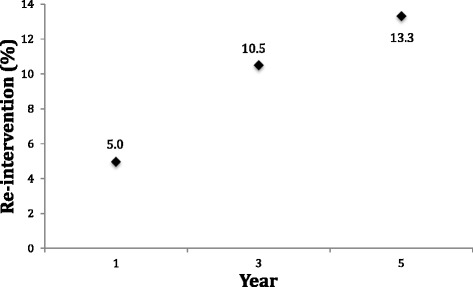
Incidence of re-intervention for all devices

Recognizing that introduction of ablation procedure types occurred at differing times, Cox proportional hazard modeling was used to assess the efficacy of each of the three methods utilized in our study. When examined from the perspective of logistic regression, being African-American increased the risk of re-operation (OR 1.40; 95%CI 1.17 to 1.67), while being in the age group of 45–49 years (OR 0.72; 95%CI 0.61 to 0.85) or ≥ 50 years (OR 0.49; 95%CI 0.37 to 0.65) significantly lowered the risk. Having a primary RFA conferred a higher likelihood of treatment failure compared to both the HTA and UBA (hazard ratio 1.37; 95%CI 1.01 to 1.86). When pathological specimens were evaluated following hysterectomy, African-American women were significantly more likely to have a diagnosis of fibroid or polyp than women identifying as Caucasian or other (*p* < .001). Survival analysis was performed using data from patients with re-intervention (*n* = 927) to evaluate for differences between endometrial ablation procedures regarding time to failure. No statistically significant differences were found (Fig. 3). Overall incidence of re-intervention was 5, 10.5, and 13.3% at year 1, 3, and 5 respectively (Fig. 4).

## Conclusions

Of the 5936 women included in the study, 927 (15.6%) underwent re-intervention with 822 (13.8%) undergoing hysterectomy and 105 (1.8%) undergoing repeat endometrial ablation. These re-intervention rates are similar to what has previously been published, with earlier large cohort studies reporting rates greater than 20%, while summaries of randomized controlled trials report rates increasing from 4.2% to over 20% based upon time from the primary procedure [3, 6].

Our study found that younger age at the time of ablation was a significant risk factor for surgical re-intervention. This risk was highest for women aged 35–39 years at the time of ablation; however, risk was increased in all women younger than 45 years. This is consistent with a majority of previous studies [4, 6, 8].

Our study did not find that indication for endometrial ablation was associated with risk of failed treatment. Menorrhagia was the most common indication for ablation in our cohort, while structural uterine anomalies (fibroid/polyp) represented the primary indication in 18% of cases. Structural uterine anomalies have been identified as risk factors for failed endometrial ablation. In the study by Wishall et al., any known structural uterine anomaly at the time of endometrial ablation was associated with hysterectomy following ablation [10]. This is believed to be due to distorted anatomy rendering the endometrial ablation less effective. Bansi-Matharu et al. published data from a retrospective review that found women with polyps present at the time of initial endometrial ablation were more likely to undergo repeat endometrial ablation [4].

Interestingly, our study identified African-American race as an independent risk factor for re-intervention following endometrial ablation. The African-American race is a known risk factor for the presence of uterine fibroids [12]. Thus, it is possible that this higher rate of treatment failure is associated with a higher incidence of uterine fibroids inherent among African-American women. This is further supported by our study, in that African-American women were significantly more likely to have fibroids or polyps on final pathology at the time of subsequent hysterectomy. Due to limitations in study design, we could not account for size, number, or location of fibroids or polyps present in women at the time of their index ablation.

The radiofrequency system conferred a higher likelihood of surgical re-intervention regardless of age, race, or procedural indication when compared to two other second-generation devices. This data differs from that of El-Nashar et al., who found in a population-based cohort study that there was no statistically significant difference in failure rates between UBA and RFA (*p* = .26) [13]. However, median follow-up in that study was 2.2 years and only included 200 patients undergoing UBA. It is possible our data are different given the larger total number of endometrial ablations, larger number of UBA, and the longer patient follow-up. It is also possible that physicians who prefer the radiofrequency device may have a lower threshold to diagnose treatment failure and surgically intervene. Based on the survival analysis, there was no statistically significant difference between the endometrial ablation devices utilized in this study with regard to time to re-intervention. Approximately half of the patients in this study failed at the 24-month period regardless of the ablation device utilized.

We found that BMI was not a risk factor for treatment failure. This is consistent with published data from Wishall et al., who performed a retrospective chart review of all endometrial ablations at Hahnemann University Hospital and the Hospital of the University of Pennsylvania from January 2006 to May 2013 [10]. Wishall found that BMI was not statistically significantly associated with treatment failure following endometrial ablation. Smithling et al. analyzed data from 968 women and had previously reported that a BMI > 30 was a statistically significant risk factor for treatment failure and re-intervention (*p* = .003) [9]. As Smithling’s data included both first- and second-generation ablation devices, it may be that the efficacy of second-generation devices can overcome risks or limitations conferred by BMI.

The overall incidence of re-intervention at years 1, 3, and 5 found in this study is lower than what has previously been published. In a large cohort study by Longinotti et al., the overall incidences of hysterectomy at years 1 and 5 were 9.3 and 22.2%, respectively [6]. That study included women undergoing both first- and second-generation endometrial ablations which could explain the lower incidences found in this study of newer second-generation ablation devices.

Due to data limitations, we are unable to comment whether tubal ligation affected long-term treatment outcome. Results regarding the effect of tubal ligation on outcome after endometrial ablation vary, with numerous reports documenting no effect [8, 14]. However, conflicting data suggest that women with a history of tubal ligation are more likely to experience treatment failure after endometrial ablation compared to women without a history of tubal ligation [5, 9]. Our study is limited in that women with a tubal ligation performed at an outside institution would not have been captured in this analysis.

Limitations of our study include those inherent in its retrospective nature. Data abstraction was dependent on procedural coding, which can be subject to bias and inaccuracies. We identified 363 (5.8%) women who had inaccurate coding regarding their initial or subsequent procedure. The potential for loss to follow-up was unavoidable as there was no way to identify women who may have had an initial endometrial ablation at the study center and had a re-intervention at an outside institution. Strengths of this study include its large sample size as well as the utilization of individual chart review to ensure accurate data abstraction. This is also the only study to date to include only second-generation ablation devices. This can impact patient counseling, as much of the existing data included first-generation ablation techniques, which have largely fallen out of favor.

We conclude that the use of second-generation endometrial ablation in the community is successful for over 80% of women. Providers should consider a patient’s age, race, and their own device preference at the time of endometrial ablation when counseling patients regarding likelihood of re-intervention. Over the last decade, it does not appear that the incidence of surgical re-intervention after endometrial ablation has significantly decreased despite evidence identifying patient risk factors. Improved patient selection could serve to lower this incidence.

## Abbreviations

CPT: Current Procedural Terminology
HTA: Hydrothermal ablation device
ICD-9: International Classification of Diseases—Ninth revision
RFA: Radiofrequency ablation
UBA: Uterine balloon ablation system
BMI: Body mass index
